# The early impact of the global lockdown on post-secondary students and staff: A global, descriptive study

**DOI:** 10.1177/20503121221074480

**Published:** 2022-01-25

**Authors:** Behdin Nowrouzi-Kia, Leeza Osipenko, Parvin Eftekhar, Nasih Othman, Sultan Alotaibi, Alexandra M Schuster, Hae Sun Suh, Andrea Duncan

**Affiliations:** 1 Department of Occupational Science and Occupational Therapy, Temerty Faculty of Medicine, University of Toronto, Toronto, ON, Canada; 2 Krembil Research Institute – University Health Network, Toronto, ON, Canada; 3 Toronto Rehabilitation Institute (KITE) – University Health Network, Toronto, ON, Canada; 4 Department of Health Policy, London School of Economics and Political Science, London, UK; 5 Cardiac Center, King Fahad Armed Forces Hospital, Jeddah, Saudi Arabia; 6 The LockedDown Project, London School of Economics and Political Science, London, UK; 7 College of Pharmacy, Kyung Hee University, Seoul, Republic of Korea

**Keywords:** COVID-19, mental health, students, lockdown, post-secondary, faculty, staff

## Abstract

**Objectives:**

The aim of this study was to gain a preliminary, broad-level understanding of how the first lockdown impacted post-secondary students, faculty, and staff worldwide.

**Methods:**

The data were obtained via a global online cross-sectional questionnaire survey using a mixed-method design and disseminated to university students, faculty, and staff from April to November 2020. The data were categorized in four themes/categories: (1) social life and relationships, (2) access to services, (3) health experiences, and (4) impact on mental health well-being.

**Results:**

The survey included 27,804 participants from 121 countries and 6 continents. The majority of participants were from Europe (73.6%), female (59.2%), under 30 years of age (64.0%), living in large urban areas (61.3%), %), and from middle-income families (66.7%). Approximately 28.4% of respondents reported that the lockdown negatively impacted their social life, while 21.2% reported the lockdown had a positive impact. A total of 39.2% reported having issues accessing products or services, including essentials, such as groceries, or medical services. In addition, respondents reported an increase in stress and anxiety levels and a decrease in quality of life during the first 2 weeks of the lockdown.

**Conclusions:**

The COVID-19 pandemic and lockdown measures had an evident impact on the lives of post-secondary students, faculty, and staff. Further research is required to inform and improve policies to support these populations at both institutional and national levels.

## Introduction

The coronavirus disease 2019 (COVID-19) is still a major public health problem despite the rapid rollout of vaccination. As of 3 December 2021, over 264 million cases have been reported worldwide to date resulting in over 5.2 million lives lost.^1^ The pandemic continues down the path no one could have imagined when the world first heard about it 2 years ago. COVID-19 first appeared in Wuhan, China, in December 2019.^2^ The disease spread rapidly and was declared a global pandemic by the World Health Organization only 3 months later.^2^ By the end of March 2020, 177 countries reported 722,435 positive cases of COVID-19, with more than 33,997 related deaths.^1^ The severity of the devastation led many federal governments to implement various measures to contain and mitigate domestic COVID-19 outbreaks, including national lockdowns.^3^

While the literature indicates the implementation of national lockdowns helped contain the spread of COVID-19,^4–11^ a growing body of evidence suggests these measures, in conjunction with the pandemic, has had adversely affected the health,^12,13^ as well as the economic and social-well-being of systems and populations at international, national, and individual levels.^7,14–16^ The pandemic has increased pressures on global supply chains, such as food and medical supplies.^17^ Additional studies have found lockdowns led to higher unemployment, poverty, and domestic violence.^18–20^ At an individual level, the pandemic and the lockdown have been linked to increased mental health stress, morbidity, and mortality.^21,22^ Other studies have reported increased rates of anxiety,^23–26^ depression,^21,22,24,25^ and suicidality.^23,25,27,28^ As a result, the COVID-19 pandemics represents unique challenges that from previous pandemics such as severe acute respiratory syndrome (SARS) and Ebola.^29^

The pandemic and lockdown have also had a notable impact on educational institutions worldwide. In modern history, before COVID-19, no comparable radical changes took place to affect the education process globally. Lockdown practices resulted in the closure of schools and post-secondary institutions, causing students and teachers to transition from in-person to online modes of education rapidly.^30^ As of 1 April 2020, 173 countries were reporting country-wide closure of all educational institutions, impacting 1.5 billion learners world-wide.^31^ It remains unclear what proportion of students and staff affected came from post-secondary institutions; regardless, researchers predict the stress of the pandemic and lockdown measures will lead to an increase in adverse psychological reactions throughout these populations.^32^

Globally, several studies have published their findings examining the socioeconomic, mental and physical health and political impacts of the COVID-19 on students ^33–37^and staff.^38^ Research has begun to emerge examining the consequences of the pandemic and lockdown on post-secondary populations at a country level. A study in Greece highlighted a significant increase in students’ mental health symptoms and suicidal ideation (*n* = 1000). In addition, 57% of students reported a decrease in quality of life.^39^ A study in Spain found subjective improvement of migraines during the lockdown was reported to worsen in 47.3% (*n* = 105) of students and concluded that university communities need to work to address these concerns.^40^

There is an emergence of research regarding the pandemic and lockdown on post-secondary populations at a country level. The aim of this study was to gain a preliminary, broad-level understanding of how the first lockdown impacted post-secondary students and staff worldwide.

## Methods

### Design

**Figure 1.**
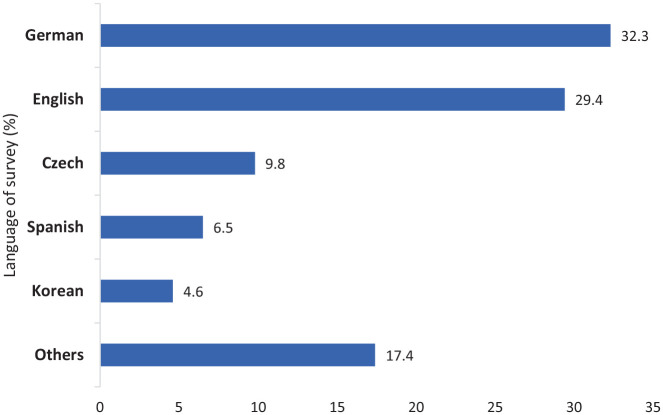
Language of survey.

The data for this study were obtained from a larger international online cross-sectional questionnaire (Supplemental Appendix—Questionnaire) using a mixed-mode design.^41,42^ The study was approved by the London School of Economics Research Ethics Review Board, the University of Toronto Research Ethics Board (#39868), and the Pusan National University Institutional Review Board (2020_62_HR). Questions of the survey focused on the impact of the global pandemic on university students, staff, and the general population. The survey was designed by researchers at the London School of Economics and piloted with 20 students and members of staff nationally and internationally. The survey was then translated into 16 languages, and each translation was validated by at least two native speakers (Figure 1).

Participants were identified as university students, faculty, staff, or as members of the generation population. A questionnaire was developed and used standard self-reported demographic, self-reported mental health, and occupational questions. The variables included age, sex and gender, employment status (e.g. part-time), residence, geographic location, family income, physical activity, social life, being in a relationship, and identification as an essential and/or key worker.

### Data collection

Universities across the world were invited to join the collaboration and help collect data in local languages. Inclusion criteria were any individual connected with a post-secondary institution; including students, faculty, and staff. In addition, responses from individuals who were not connected with a post-secondary institution were collected but segregated for purposes of analysis. The questionnaire was launched on 22 April 2020 and closed on 21 November 2020. The questionnaire was available through the following website: https://www.healthbit.com/the-lockeddown. For the quantitative portion of the questionnaire (including all questions except the last one), data capture was structured in such a way that the language in which the survey is filled out does not prevent any problems with data compilation and analysis.

### Statistical analysis

The descriptive analyses were performed in STATA version 13.0.^43^ Of the total 30,532 records obtained from the online survey, there were 857 (2.8%) failed attempts (blank records) where only unique ID and date were present (automatically generated), indicating an attempt without taking the survey. These observations were excluded from the study and deleted, leaving 29,675 observations. Checking for duplicates showed that there were 2,049 duplicates (1871 surplus, 6.3%). These surplus observations were deleted, leaving 27,804 observations for analysis. Duplicate observations were found in all languages, and included little data having the majority of the variables missing. Apart from age, all variables were categorical. Missing values of age were imputed with the median value of age separately for each category of students, faculty, staff, and non-affiliated participants. Bivariate analysis for self-reported stress level was done using chi-square test to assess association of increased stress level with several demographic and other theoretically plausible variables.

## Results

The survey had a total of 27,804 participants across 121 countries and 6 continents, with 93% of responses collected between May and July of 2020. Participants included 17,258 students, 7843 university staff, and 3052 individuals unaffiliated with any post-secondary institution (see Table 1). Majority of participants were from Europe (73.6%), female (59.2%), under 30 years of age (64.0%), living in large urban areas (61.3%), and from middle-income families (66.7%). The age of participants ranged from 17 to 99, with a mean age of 30.5 years (standard deviation (SD) 12.6). Approximately 19.2% of respondents reported having a chronic health condition. See Table 1 and Figure 1 for more details.

**Table 1.** Demographic characteristics of respondents.^1^

Characteristics		Number	Percent (%)
All		27,804	100
Category	University staff	7843	26.9
	University students	17,258	62.1
	Not affiliated with a university	3062	11.0
Month of survey	April	802	2.9
	May	2623	9.4
	June	10,200	36.7
	July	13,120	47.2
	August–November	1059	3.8
Continent	Asia	4120	14.9
	Africa	239	0.9
	Europe	19,794	71.4
	North America	1085	3.9
	South America	2275	8.2
	Oceana	208	0.7
Age group	Under 30	17,788	64.0
	30–49	6852	24.6
	50 and over	3164	11.4
Gender	Female	15,923	59.2
	Male	10,500	39.1
	Other	116	0.4
	Prefer not to say	349	1.3
Residence	Large city	16,785	61.3
	Small city/town	7160	26.1
	Countryside/suburb	3443	12.6
Family income level	High income	3687	13.7
	Low income	3329	12.4
	Middle income	17,940	66.7
	Prefer not to say	1924	7.2
Age in years, mean (SD)		30.5 (12.6)	

SD: standard deviation.

1 Complete case analysis is done, missing values are not included in the table.

### Social life, relationships, and activities during the lockdown/pandemic

The survey had 10 questions on the impact of the lockdown on social life, relationships, and other activities. In relation to social life, 21.2% of respondents described their social life as great, while 28.4% said their social life was negatively affected due to the lockdown. The remaining half of respondents reported that they managed to cope with the changes to their social life. In total, 57.3% of respondents reported that the lockdown had no effect on their relationships, while 23.3% reported that their relationships suffered or fell apart. Exercising habits were also affected due to the lockdown. Approximately 42% of respondents said they were not able to exercise as before or at all, while 37% said they could do sufficient or even more exercise (Table 2).

**Table 2.** Reported social life and related activities during the first lockdown.

Characteristics		Number	Percent (%)
Social life	Has been great and I managed to stay positive	5480	21.2
	Was impacted but overall I am/was able to cope owing to other support	12,992	50.4
	Was negatively impacted	7316	28.4
Relationship with partner	Improved	3169	19.4
	Was not affected	9363	57.3
	Suffered/fell apart	3810	23.3
I have/had a troubled relationship with people I live with		4556	20.1
Exercise	Don’t exercise, no change for me	5404	21.0
	Do sufficient/more exercise	9543	37.0
	Couldn’t exercise as before	10,839	42.0
I was responsible for childcare		3904	17.2
	Childcare significantly impacted my education/work	2159	55.3
I was a carer for a sick person		1662	6.4

### Access to services

**Figure 2.**
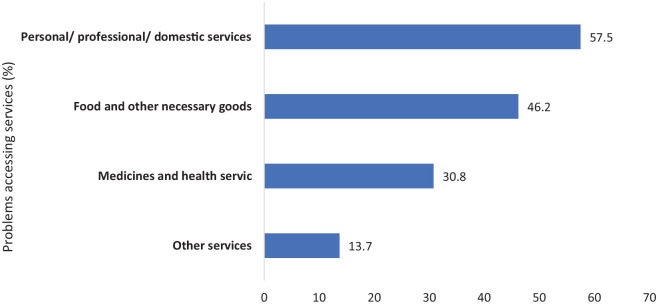
Participants who reported having problems accessing services during the first lockdown.

A total of 10,110 (39.2%) respondents reported having issues accessing products or services. Of those who reported problems, 46.2% mentioned troubles accessing food and other necessary goods, 57.5% reported problems accessing personal/professional/domestic services, 30.8% mentioned issues accessing medicines and health services, while 13.7% reported problems accessing goods or services outside the scope of answers (see Figure 2).

### Health experiences

Table 3 summarizes health-related experiences of participants. Just over 19% of respondents reported having an underlying health condition. In relation to COVID-19, 10.1% reported experiencing COVID-19 symptoms, and 2.3% reported losing someone close to the virus. Over 28% of respondents reported experiencing a non-COVID-19 health issue during the lockdown. Approximately 3.9% of respondents reported losing someone close to them due to a non-COVID-19-related health condition. Respondents also reported issues accessing healthcare services during the pandemic. Approximately 18% of respondents reported not being able to access the health services effectively. In addition, 10.4% of respondents reported that someone in their family experienced a health emergency which was not adequately dealt with during the pandemic.

**Table 3.** Reported health experiences during the first lockdown.

Condition	Number	Percent (%)
Has underlying health condition	5164	19.2
Had non-COVID-related health issues	7243	28.4
Was not able to effectively access health services	4564	17.9
Lost someone close to COVID-19	596	2.3
Lost someone close to another health condition	993	3.9
Someone in my family had a health emergency but not adequately dealt with	2643	10.4
I had COVID-19 symptoms	2583	10.1
I was tested	611	23.7
My test result was positive	497	81.3
My test results was negative	87	14.3
My test result was not provided	27	4.4

### Impact on mental well-being

**Figure 3.**
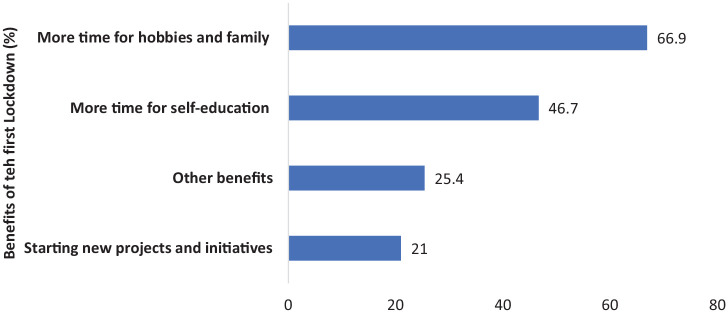
Reported benefits of the first lockdown.

Participants were asked about their stress level, anxiety, and quality of life during the lockdown. As shown in Figure 3, a considerable proportion of respondents reported a worsening of these conditions, especially during the first 2 weeks of the lockdown. For example, 46.7%, 37.8%, and 36.5% reported increased levels of stress during week 1–2, week 3–4, and week 5 of the lockdown, respectively. Similarly, 41.9%, 39.5%, and 34.8% of respondents reported that their quality of life decreased during the same three periods.

**Figure 4.**
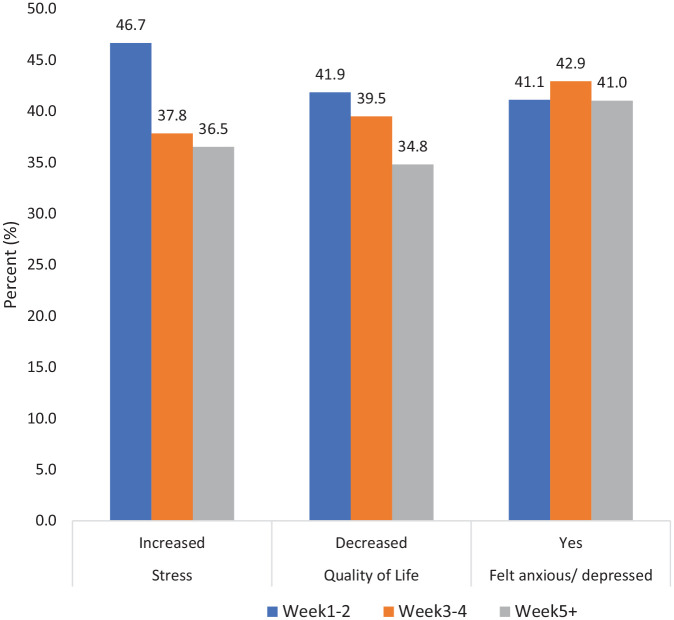
Self-reported anxiety, stress, and quality of life during the first lockdown.

There was a statistically significant association between changes in stress level with all variables shown in Table 4 and Figure 4. Those who reported the greatest increases in stress levels throughout the lockdown included respondents who: self-identified their gender as ‘other’; belonged to younger age groups; lived in a larger urban area; could not exercise as before; reported the lockdown negatively impacted their social life; reported that their relationships suffered; continued to work as an essential worker (key worker); had underlying health condition(s); and came from low-income families. In relation to reported changes in quality of life, the statistically significant associations are shown in Table 5. Respondents who reported the greatest decrease in quality of life were respondents who: self-identified as their gender as other; belonged to younger age groups; lived in large urban area; came from low-income families; reported the pandemic and lockdown negatively impacted their social life or reported that their relationships suffered; could not exercise as before; and had underlying health condition(s).

**Table 4.** Changes to self-reported stress levels during the first lockdown by demographic, social, and health-related factors.

Characteristics		Stress level			*χ* ^2^_*p*_
		Decreased number (%)	Increased number (%)	Same number (%)	
Category	University staff	1517 (22.2)	2422 (35.4)	2904 (42.4)	49.3 < 0.001
	University students	2998 (19.3)	6003 (38.7)	6518 (42.0)	
	Not affiliated	436 (16.9)	1016 (39.3)	1133 (43.8)	
Continent	Asia	386 (11.4)	1519 (44.7)	1492 (43.9)	322.6 < 0.001
	Africa	27 (13.9)	92 (47.4)	75 (38.7)	
	Europe	4010 (22.0)	6423 (35.3)	7779 (42.7)	
	North America	177 (17.8)	444 (44.6)	374 (37.6)	
	South America	315 (16.2)	887 (45.5)	749 (38.4)	
	Oceana	36 (18.2)	76 (38.4)	86 (43.4)	
Age group	Under 30	3151 (19.8)	6082 (38.1)	6713 (42.1)	99.6 < 0.001
	30–49	1249 (20.5)	2459 (40.3)	2398(39.3)	
	50 and over	551 (19.0)	900 (31.1)	1444 (49.9)	
Gender	Female	3028 (20.4)	5921 (39.9)	5908 (39.8)	156.4 < 0.001
	Male	1868 (19.3)	3307 (34.2)	4508 (46.6)	
	Other	14 (12.6)	62 (55.9)	35 (31.5)	
	Prefer not to say	41 (13.9)	151 (55.9)	104 (35.1)	
Residence	Large city	3010 (19.8)	5837 (38.3)	6387 (41.9)	39.8 < 0.001
	Small city/town	1210 (18.5)	2531 (38.8)	2784 (42.7)	
	Countryside/suburb	731 (22.9)	1073 (33.7)	1384 (43.4)	
Family income level	High income	762 (22.0)	1180 (34.1)	1522 (43.9)	99.7 < 0.001
	Low income	548 (17.8)	1380 (44.9)	1144 (37.2)	
	Middle income	3338 (20.0)	6280 (37.5)	7116 (42.5)	
	Prefer not to say	303 (18.1)	601 (35.8)	773 (46.1)	
Social life	Has been great	1374 (26.6)	998 (19.4)	2785 (54.0)	1900.0 < 0.001
	Was impacted but I cope	2715 (21.5)	4380 (34.7)	5540 (43.9)	
	Was negatively impacted	862 (12.1)	4063 (56.8)	2230 (31.2)	
Relationship with partner	Improved	940 (30.4)	901(29.1)	1253 (40.5)	687.6 < 0.001
	Was not affected	1695 (18.4)	3122 (34.7)	4180 (46.5)	
	Suffered/fell apart	545 (14.6)	1999 (53.5)	1192 (31.9)	
Exercise	Do not exercise, no change	876 (16.9)	1960 (37.8)	2351 (45.3)	503.5 < 0.001
	Do sufficient/more	2319 (25.1)	2813 (30.5)	4099 (44.4)	
	Could not do as before	1756 (16.7)	4668 (44.3)	4105 (39.0)	
Worked as a keyworker during lockdown/pandemic		621 (18.9)	1317 (40.0)	1356 (41.2)	7.6, 0.02
Has underlying health condition		922 (16.1)	2100 (43.6)	1800 (37.3)	88.1 < 0.001

**Table 5.** Change to self-reported quality of life during the first lockdown by demographic, social, and health-related factors.

Characteristics		Stress level			*χ* ^2^_*p*_
		Decreased number (%)	Increased number (%)	Same number (%)	
Category	University staff	2502 (36.3)	986 (14.3)	3414 (49.5)	60.1, <0.001
	University students	6428 (41.0)	1819 (11.6)	7422 (47.4)	
	Not affiliated	1016 (38.9)	332 (12.7)	1263 (48.4)	
Continent	Asia	1418 (41.1)	394 (11.4)	1635 (47.4)	168.9, <0.001
	Africa	81 (41.8)	31 (16.0)	82 (42.3)	
	Europe	7366 (40.2)	2385 (13.0)	8583 (46.8)	
	North America	411 (41.1)	115 (11.5)	473 (47.4)	
	South America	585 (29.1)	191 (9.5)	1236 (61.4)	
	Oceana	85 (43.4)	21 (10.7)	90 (45.9)	
Age group	Under 30	6525 (40.5)	1903 (11.8)	7671 (47.7)	84.1, <0.001
	30–49	2380 (38.8)	915 (14.9)	2842 (46.3)	
	50 and over	1041 (35.3)	319 (10.8)	1586 (53.8)	
Gender	Female	5948 (39.7)	1933 (12.9)	7088 (47.4)	21.5. 0.002
	Male	3816 (38.9)	1153 (11.8)	4841 (49.4)	
	Other	57 (51.4)	11 (9.9)	43 (38.7)	
	Prefer not to say	125 (42.8)	40 (13.7)	127 (43.5)	
Residence	Large city	6243 (40.6)	1854 (12.1)	7289 (47.4)	40.3, <0.001
	Small city/town	2573 (39.5)	809 (12.3)	3026 (48.7)	
	Countryside/suburb	1130 (35.2)	474 (14.8)	1604 (50.0)	
Family income level	High income	1331 (37.9)	530 (15.1)	1649 (47.0)	154.2, <0.001
	Low income	1506 (48.7)	339 (11.0)	1247 (40.3)	
	Middle income	6481 (38.4)	2076 (12.3)	8317 (49.3)	
	Prefer not to say	628 (36.8)	192 (11.3)	886 (51.9)	
Social life	Has been great	834 (15.7)	1164 (22.0)	3301 (62.3)	3300, <0.001
	Was impacted but I cope	4493 (35.4)	1502 (11.8)	6708 (52.8)	
	Was negatively impacted	4619 (64.3)	471 (6.6)	2090 (29.1)	
Relationship with partner	Improved	910 (29.4)	760 (24.5)	1430 (46.1)	960.7, <0.001
	Was not affected	3210 (35.2)	1025 (11.2)	4881 (53.4)	
	Suffered/fell apart	2095 (56.0)	315 (8.4)	1329 (35.5)	
Exercise	Do not exercise, no change	1898 (36.2)	512 (9.8)	2839 (54.1)	974.1, <0.001
	Do sufficient/more	2822 (30.3)	1662 (17.8)	4839 (51.9)	
	Could not do as before	5226 (49.3)	963 (9.1)	4421 (41.7)	
Worked as a keyworker during lockdown/pandemic		1314 (39.5)	375 (11.3)	1638 (49.2)	5.5, 0.07
Has underlying health condition		2150 (44.2)	579 (11.9)	2139 (43.9)	56.0, <0.001

### Benefits of the lockdown

A total of 8127 (31.5%) respondents said that the lockdown was beneficial to them. Benefits included more time for hobbies and family (66.9%), self-education (46.7%), or new projects and initiatives (21.0%). A total of 25.4% respondents reported other benefits.

## Discussion

We examined the impact of the lockdown on the mental health of participants from post-secondary education settings around the world. Specifically, to study the demographic and mental health of students, faculty, and staff across 121 countries and 6 continents. This initiative was a unique opportunity to assess in a short time frame, the impact of the lockdown and physical distancing on the global population of students, faculty and staff; and to inform policy-makers and educational institutions and enable them to respond relying on factual data. We sought to understand the initial impact of lockdown measures experienced by the students and staff at post-secondary institutions around the world. Consistent with other studies,^22,32,39,44,45^ a cohort of respondents reported an overall increase in stress and decreasedquality of life over the first 5 weeks of the lockdown. Students and staff reported similar changes to stress and quality of life. Approximately, a third of participants reported that the spring/summer 2020 lockdown was beneficial to them, as it allowed them to dedicate more time to family, hobbies, projects, and self-directed initiatives. Studies suggest that during pandemics, communication can play a significant role in reducing apprehension and uncertainty while promoting a unified fight against public health threats.^15^

Regarding socialization, most respondents in the study reported they could maintain a social life during lockdown, or cope with the changes to their social lives that resulted from lockdown. In contrast, a third of respondents reported that their social life was negatively affected. For respondents who took part in exercise prior to the lockdown, there was an even split between those who reported that they could do more exercise, and those who reported they could not exercise as they did before.

This study had a strong response rate which supported statistical analysis and will allow for future analysis of various subgroups. Most of the responses were received from German, English, Czech, Spanish, and Korean language surveys. However, we acknowledge that these results were weighted to respondents from European countries.

Due to the interest in launching this survey rapidly, there was limited testing of validity and reliability of the questions. Standardized tools were not used, and there was a lack of operational definitions for key constructs. Moreover, we did not control for the temporality of the responses (e.g. responses were collected between May and November 2020). Therefore, how participants have reported their own stress, quality of life, and mental health may have varied significantly. Specifically, while some respondents reported being significantly impacted by the lockdown, others highlighted how it impacted their health in a positive manner. Finally, this was an exploratory study, and therefore, we did not perform a sample size calculation.

### Implications

The implications of this study are two-fold. First, it reinforces the postulates of previous authors that post-secondary institutions must ensure there is adequate support available for students and staff who are struggling with their health and well-being and adds to the growing body regarding the impacts of the lockdown on post-secondary students health.^4,46–48^ Second, it leads to questions about how post-secondary institutions identify who is in need of more support, so their outreach can be timely and targeted. Specifically, the findings highlight that universities need to develop practices and approaches to address emerging needs when a significant public health crisis occurs. Finally, the study provides preliminary evidence regarding the impacts of the first lockdown on students, faculty, and staff including their health and well-being.

## Conclusion

The pandemic has negatively impacted the social and quality of life of post-secondary students and staff globally. This situation affected their productivity and access to services. Further quantitative and qualitative studies to explore the depth of COVID-19 are required to examine the effect and problems to plan public health policies and inform social and health care outreach initiatives.

Many surveys were undertaken during the start of the pandemic, and there is a need for comparative analyses of these different findings to better map the impact of the lockdown on the academic population and other groups.

## Supplemental Material

sj-docx-1-smo-10.1177_20503121221074480 – Supplemental material for The early impact of the global lockdown on post-secondary students and staff: A global, descriptive study Click here for additional data file. Supplemental material, sj-docx-1-smo-10.1177_20503121221074480 for The early impact of the global lockdown on post-secondary students and staff: A global, descriptive study by Behdin Nowrouzi-Kia, Leeza Osipenko, Parvin Eftekhar, Nasih Othman, Sultan Alotaibi, Alexandra M Schuster, Hae Sun Suh and Andrea Duncan in SAGE Open Medicine
