# Supplementary material for: The early impact of the global lockdown on post-secondary students and staff: A global, descriptive study
Source: SAGE Open Med. 2022 Jan 25;10:20503121221074480. doi: 10.1177/20503121221074480 (PMC8793123; doi:10.1177/20503121221074480)
Supplement: sj-docx-1-smo-10.1177_20503121221074480 – Supplemental material for The early impact of the global lockdown on post-secondary students and staff: A global, descriptive study [file sj-docx-1-smo-10.1177_20503121221074480.docx]

| QUESTION | **ENGLISH TERM** | CONTEXT | RESPONSES | **ADD TRANSLATION HERE** |
| --- | --- | --- | --- | --- |
|  | The LockedDown | Title |  |  |
|  | The pandemic altered everyonès lives. Whether you ended up in lockdown or in isolation, continued to work or stopped studying, the world’s focus on Covid-19 does not make you invisible or unaccounted. Your experience matters, unlock it to the world so we can construct a better factual map of what is happening to every one of us in 2020. Your story will be heard.  Learn more About the project.   - The survey is anonymised and will take 7-10 minutes. - All data collected is GDPR compliant and will be used for research purposes only. | Introduction text |  |  |
|  | **Consent**  **I am over 17 years of age and I understand that data I’m providing will be used for research purposes only. I understand that this survey is anonymised and no personal data will be collected to establish my identity.**  Should you be negatively impacted by any of the questions in this survey, links to sources of support are provided at the end. |  |  |  |
|  | I Understand | Tick-box consent |  |  |
|  | I’m a: |  |  |  |
|  |  |  | Please choose… |  |
|  |  |  | Student at a university |  |
|  |  |  | Staff at a university |  |
|  |  |  | Not affiliated with a university |  |
|  | Type: |  |  |  |
|  |  |  | Graduate |  |
|  |  |  | Undergraduate |  |
|  | Type: |  |  |  |
|  |  |  | Academic |  |
|  |  |  | Non-academic |  |
|  | Type: |  |  |  |
|  |  |  | Full time |  |
|  |  |  | Part-time |  |
|  | Which country is your University in? |  |  |  |
|  |  |  | Please choose... |  |
|  | University name: |  |  |  |
|  |  |  | Please choose... |  |
|  | University name: |  |  |  |
|  | If no drop-down option available | Sub-text to help you to use this field if no -drop-lists are presented. |  |  |
| 1 | Country of residence |  |  |  |
| 2 | I live in: |  |  |  |
|  |  |  | Large city |  |
|  |  |  | Small city / town |  |
|  |  |  | Countryside / suburb |  |
| 3 | My accommodation is a: |  |  |  |
|  |  |  | Flat |  |
|  |  |  | House |  |
|  |  |  | Rented room |  |
|  | Tell us about yourself | Title |  |  |
| 4 | I am: |  |  |  |
|  |  |  | Employed |  |
|  |  |  | Not Employed |  |
|  |  |  | Retired |  |
| 5 | My occupation |  |  |  |
| 6 | Age: (Enter number like 24 not DOB) |  |  |  |
|  | Enter your age as a number e.g. 24 | Placeholder inside field |  |  |
| 7 | Gender: |  |  |  |
|  |  |  | Male |  |
|  |  |  | Female |  |
|  |  |  | Prefer not to say |  |
|  |  |  | Other |  |
| 8 | I am / was (prior to the pandemic) employed: |  |  |  |
|  |  |  | Yes |  |
|  |  |  | No |  |
| 9 | Full-time or part-time employment: |  |  |  |
|  |  |  | Full-time |  |
|  |  |  | Part-time |  |
| 10 | My family is: |  |  |  |
|  |  |  | Low income |  |
|  |  |  | Middle Income |  |
|  |  |  | High Income |  |
|  |  |  | Prefer not to say |  |
| 11 | I have underlying health condition(s) : |  |  |  |
|  |  |  | Yes |  |
|  |  |  | No |  |
| 12 | I continued to work as a key worker (e.g. hospital staff, security, etc) during the pandemic |  |  |  |
|  |  |  | Yes |  |
|  |  |  | No |  |
|  | Please progress with the survey as all questions still apply and your answers will help us understand how you are/were affected by the pandemic. | If Yes selected, information displayed. |  |  |
|  | Professional Life | Title |  |  |
| 13 | I'm a business owner / self-employed / entrepreneur |  |  |  |
|  |  |  | Yes |  |
|  |  |  | No |  |
| 13a | You can select multiple options. |  |  |  |
|  |  |  | My business is not impacted by the pandemic |  |
|  |  |  | I may lose my business as a result of the lockdown/pandemic |  |
|  |  |  | My business was negatively impacted |  |
|  |  |  | My business was positively impacted |  |
|  |  |  | I may go bankrupt |  |
|  |  |  | I need financial support from the government |  |
| 14 | My lockdown status |  |  |  |
|  |  |  | I'm/**was** in lockdown |  |
|  |  |  | I continue/ed to work as a key worker |  |
|  |  |  | Not applicable |  |
| 14a | Since the start of the pandemic: |  |  |  |
|  |  |  | I am/was unable to work but I keep/kept getting paid |  |
|  |  |  | I am/was unable to work and I lost / am losing my income |  |
|  |  |  | I continue/ed to work virtually - not much difference besides a different setting |  |
|  |  |  | I continue/ed to work virtually but it is not going well |  |
|  |  |  | I worked from home anyway, there was no change for me |  |
| 15 | I am/have been anxious I might get COVID and/or infect my family |  |  |  |
|  |  |  | Yes |  |
|  |  |  | No |  |
| 16 | My work became more intense and stressful because of COVID |  |  |  |
|  |  |  | Yes |  |
|  |  |  | No |  |
| 17 | I am / my family are experiencing financial difficulties due to lockdown/pandemic: |  |  |  |
|  |  |  | Yes |  |
|  |  |  | No |  |
| 18 | I lost my job / part-time employment due to lockdown/pandemic: |  |  |  |
|  |  |  | Yes |  |
|  |  |  | No |  |
| 19 | I'm anxious about my job security: |  |  |  |
|  |  |  | Yes |  |
|  |  |  | No |  |
| 20 | I need support (social services, psychological, physical, etc) and I don't have any or some of it: |  |  |  |
|  |  |  | Yes |  |
|  |  |  | No |  |
| 21 | I need support and I'm getting adequate support: |  |  |  |
|  |  |  | Yes |  |
|  |  |  | No |  |
|  | Wellbeing | Title |  |  |
| 22 | Right before or once the lockdown had been introduced (the pandemic announced) due to Covid 19: |  |  |  |
|  |  |  | I moved in with my parents |  |
|  |  |  | I remained in my residency |  |
|  |  |  | I moved elsewhere |  |
|  |  |  | I was unable to travel to be where I wanted to be during lockdown |  |
| 22a | Which country did you move to? |  |  |  |
|  |  |  | Please choose... |  |
| 23 | Due to the lockdown / Covid-19 pandemic: |  |  |  |
|  |  |  | My social life was negatively impacted |  |
|  |  |  | My social life was impacted but overall I am/was able to cope owing to other support |  |
|  |  |  | My social life has been great and I managed to stay positive |  |
| 24 | Due to the lockdown / Covid-19 pandemic my relationship with my partner: |  |  |  |
|  |  |  | Suffered |  |
|  |  |  | Fell apart |  |
|  |  |  | Was not affected |  |
|  |  |  | Improved |  |
|  |  |  | I'm not in a relationship |  |
| 25 | During lockdown/pandemic I had problems accessing products or services (physically or online): |  |  |  |
|  |  |  | Yes |  |
|  |  |  | No |  |
| 25a | I had problems accessing: |  |  |  |
|  | You can select multiple options. |  | Personal/Professional/Domestic services |  |
|  |  |  | Medicines/Health services |  |
|  |  |  | Food and other necessary goods |  |
|  |  |  | Other |  |
| 26 | I'm /was in lockdown with: |  |  |  |
|  |  |  | I'm alone |  |
|  |  |  | Housemates |  |
|  |  |  | Partner |  |
|  |  |  | My parents (and siblings) |  |
|  |  |  | My family (and children) |  |
|  |  |  | Other |  |
| 27 | I am a carer / I cared for a sick person in lockdown/during the pandemic: |  |  |  |
|  |  |  | Yes |  |
|  |  |  | No |  |
| 28 | Exercise during lockdown/pandemic: |  |  |  |
|  |  |  | I started to exercise more |  |
|  |  |  | I get sufficient exercise and I'm satisfied |  |
|  |  |  | I can exercise but it is not how I want it |  |
|  |  |  | I don't exercise, no change for me |  |
|  |  |  | I cannot exercise but it does not bother me |  |
|  |  |  | I cannot exercise and it decreases my quality of life |  |
| 29 | I am a professional athlete (university team): |  |  |  |
|  |  |  | Yes |  |
|  |  |  | No |  |
| 29a | I'm anxious lockdown/pandemic impacts my athletic career/performance: |  |  |  |
|  |  |  | Yes |  |
|  |  |  | No |  |
| 30 | In lockdown / during pandemic, my level of stress: |  |  |  |
|  |  |  | **Increased** |  |
|  |  |  | **Decreased** |  |
|  |  |  | **Stayed the same** |  |
|  |  |  | **Not applicable** |  |
|  | **Week 1-2** |  |  |  |
|  | **Week 3-4** |  |  |  |
|  | **Week 5+** |  |  |  |
| 31 | In lockdown / during pandemic, my quality of life: |  |  |  |
|  |  |  | **Increased** |  |
|  |  |  | **Decreased** |  |
|  |  |  | **Stayed the same** |  |
|  |  |  | **Not applicable** |  |
|  | **Week 1-2** |  |  |  |
|  | **Week 3-4** |  |  |  |
|  | **Week 5+** |  |  |  |
| 32 | In lockdown / during pandemic, I feel/felt depressed/anxious: |  |  |  |
|  |  |  | Yes |  |
|  |  |  | No |  |
|  |  |  | Not applicable |  |
|  | **Week 1-2** |  |  |  |
|  | **Week 3-4** |  |  |  |
|  | **Week 5+** |  |  |  |
|  |  |  |  |  |
|  | **Lockdown is/was beneficial for me:** | Title |  |  |
|  |  |  | Yes |  |
|  |  |  | No |  |
|  |  |  | Not applicable |  |
| 33 | Why is it/has it been beneficial? |  |  |  |
|  | You can select more than one. |  |  |  |
|  |  |  | I have/had more time for my hobbies /family/ relationship |  |
|  |  |  | I dedicate/-ed more time to self-education |  |
|  |  |  | I started/joined new projects/initiatives/jobs (paid/unpaid) |  |
|  |  |  | Other |  |
| 34 | In lockdown/during the pandemic I have/had a troubled relationship with people I live with: |  |  |  |
|  |  |  | Yes |  |
|  |  |  | No |  |
| 35 | In lockdown/ during the pandemic I experience/-ed domestic abuse (psychological abuse): |  |  |  |
|  |  |  | Yes |  |
|  |  |  | No |  |
| 36 | I'm/was responsible for childcare in lockdown/ during the pandemic: |  |  |  |
|  |  |  | Yes |  |
|  |  |  | No |  |
| 36a | Childcare significantly impacted my education/work: |  |  |  |
|  |  |  | Yes |  |
|  |  |  | No |  |
|  | Health | Title |  |  |
| 37 | COVID-19 Symptoms: |  |  |  |
|  |  |  | I had covid-19 symptoms but was not tested for Covid |  |
|  |  |  | I had covid-19 symptoms and was tested for Covid |  |
|  |  |  | I had covid-19 symptoms and was refused the test |  |
|  |  |  | I did not have covid-19 symptoms |  |
| 38 | My COVID-19 Test Result: |  |  |  |
|  |  |  | Positive |  |
|  |  |  | Negative |  |
|  |  |  | Results not provided |  |
| 39 | In Lockdown/during the pandemic: |  |  |  |
|  | I had/have non-covid related health issues: |  |  |  |
|  |  |  | Yes |  |
|  |  |  | No |  |
| 40 | I was effectively able to access health services: |  |  |  |
|  |  |  | Yes |  |
|  |  |  | No |  |
|  |  |  | Not applicable |  |
|  | Since the start of the pandemic: | Title |  |  |
| 41a | I lost someone close to me due to Covid-19: |  |  |  |
|  |  |  | Yes |  |
|  |  |  | No |  |
| 41b | I lost someone close to me due to another health condition as a cause of lockdown: |  |  |  |
|  |  |  | Yes |  |
|  |  |  | No |  |
| 41c | Someone in my family experienced a health emergency, which was not adequately dealt with due to lockdown/pandemic: |  |  |  |
|  |  |  | Yes |  |
|  |  |  | No |  |
| 42 | I have a disability/chronic illness: |  |  |  |
|  |  |  | Yes |  |
|  |  |  | No |  |
| 42a | My support diminished because of the pandemic: |  |  |  |
|  |  |  | Yes |  |
|  |  |  | No |  |
| 43 | Share any other thoughts about the impact of the lockdown/pandemic on your life: |  |  |  |
|  |  |  |  |  |
|  | **Education/Work** | Title |  |  |
|  | After the University Closed: | Title |  |  |
| 44 | Teaching / Learning |  |  |  |
|  |  |  | I continued teaching / learning online |  |
|  |  |  | Continuing teaching / learning online was not possible |  |
|  |  |  | Not applicable |  |
| 45 | I was unable to continue my university work partially or fully (e.g. lab shut down, international station required) |  |  |  |
|  |  |  | Yes |  |
|  |  |  | No |  |
|  |  |  | Not applicable |  |
| 46 | My university progressed with the exams / assessments and made relevant arrangements: |  |  |  |
|  |  |  | Yes |  |
|  |  |  | No |  |
|  |  |  | Not applicable |  |
| 47 | Exams were postponed / cancelled: |  |  |  |
|  |  |  | Yes |  |
|  |  |  | No |  |
|  |  |  | Not applicable |  |
| 48 | My university was supportive in offering services which enabled me to continue my work/education: |  |  |  |
|  |  |  | Yes |  |
|  |  |  | No |  |
| 49 | I am experiencing financial difficulties due to lockdown/pandemic: |  |  |  |
|  |  |  | Yes |  |
|  |  |  | No |  |
| 50 | I lost my job/part time employment due to lockdown/pandemic: |  |  |  |
|  |  |  | Yes |  |
|  |  |  | No |  |
|  |  |  | Not applicable |  |
| 51 | At least some of my research work is now Covid related: |  |  |  |
|  |  |  | Yes |  |
|  |  |  | No |  |
| 52 | I am a special needs student and I require support: |  |  |  |
|  |  |  | Yes |  |
|  |  |  | No |  |
| 52a | About my support: |  |  |  |
|  |  |  | I lost this support due to lockdown/pandemic |  |
|  |  |  | My support was significantly impacted |  |
|  |  |  | My support was continued / no impact |  |
| 53 | My professional/educational experience was: |  |  |  |
|  |  |  | Negatively impacted |  |
|  |  |  | Positively impacted |  |
|  |  |  | Not impacted |  |
| 54 | Because of lockdown/pandemic I will not be able to continue my education in the near term (after life goes back to normal): |  |  |  |
|  |  |  | Yes |  |
|  |  |  | No |  |
| 55 | I'm anxious about my job/job security: |  |  |  |
|  |  |  | Yes |  |
|  |  |  | No |  |
| 56 | Online learning / teaching: | Title |  |  |
|  |  |  | Online learning/teaching is possible but in person it is better |  |
|  |  |  | Online learning/teaching is great and should continue |  |
|  |  |  | Online learning/teaching was not a good experience for me |  |
|  |  |  | Not applicable |  |
| 57 |  |  |  |  |
|  | You can select multiple options. |  |  |  |
|  |  |  | I may not be able to finance my education due to parents' job loss, inability to secure a loan, etc |  |
|  |  |  | I'm anxious about my education/exams |  |
|  |  |  | I'm anxious I will not be able to find a job in the near term |  |
|  |  |  | I will experience a delay in finding a job/starting new employment |  |
| 58 | I'm anxious I will not be able to secure funding for my projects (research): |  |  |  |
|  |  |  | Yes |  |
|  |  |  | No |  |
|  |  |  | Not applicable |  |
| 59 | I'm graduating and actively applying for jobs: |  |  |  |
|  |  |  | Yes |  |
|  |  |  | No |  |
| 60 | Are you still applying? |  |  |  |
|  |  |  | Yes |  |
|  |  |  | No |  |
| 61 | Receiving job offers? |  |  |  |
|  |  |  | Yes |  |
|  |  |  | No |  |
| 62 | Receiving job rejections (explicitly because of COVID-19)? |  |  |  |
|  |  |  | Yes |  |
|  |  |  | No |  |
| 63 | I'm applying/have applied for another degree program to continue my education in 2020/2021: |  |  |  |
|  |  |  | Yes |  |
|  |  |  | No |  |
| 64 | Was your plan to study abroad? |  |  |  |
|  |  |  | Yes |  |
|  |  |  | No |  |
| 64a | Which country? |  |  |  |
|  | Please choose... |  |  |  |
|  |  |  |  |  |
| 65 | Are you still considering going abroad if accepted? |  |  |  |
|  |  |  | Yes |  |
|  |  |  | No |  |
| 66 | I stopped applying/decided to wait/reluctant to go abroad. |  |  |  |
|  |  |  | Yes |  |
|  |  |  | No |  |
| 67 | Are you aware of at least one student or a member of staff who is in significant distress and as a result would be unable to take part in a survey like this? |  |  |  |
|  |  |  | Yes |  |
|  |  |  | No |  |
| 68 | Share any other thoughts / experiences about your life in lockdown/during the pandemic: |  |  |  |
|  |  |  |  |  |
|  | Thank you for sharing your experience during the pandemic. |  |  |  |
|  | Share 'The LockedDown' <https://www.healthbit.com/the-lockeddown/>  and help others share their experience too! If yoùd like to be notified of the results from this research, you can [add your email here](https://research.healthbit.com/c/subscribe). |  |  |  |
|  | If you experience distress during this difficult time, please consider seeking help from these organisations: [MIND](https://www.mind.org.uk/information-support/tips-for-everyday-living/wellbeing/wellbeing/), [Inspire Wellbeing](https://www.inspirewellbeing.org/), [LifeWorks.](https://www.lifeworks.com/) Your university wellbeing support center may also offer helpful resources. |  |  |  |
|  | Before you go - please use the Healthbit App to accelerate health research. Maintain all your health information in one place so it's easily shareable in an emergency. |  |  |  |
|  |  |  |  |  |
|  | Track all your symptoms, including common COVID-19 symptoms, and help global research. |  |  |  |
|  |  |  |  |  |
|  | [Download the Healthbit App Here](https://www.healthbit.com/get-app/) |  |  |  |
|  |  |  |  |  |
|  | Take the survey |  |  |  |
|  | Next |  |  |  |
|  | Back |  |  |  |
|  | Finish |  |  |  |
|  | Thank you for your participation! |  |  |  |
|  | Please notify me when The LockDown findings are available: | Title |  |  |
|  | Enter your email address |  |  |  |
